# Supplementary material for: Identification and Characterization of Glucosyltransferase That Forms 1-Galloyl-β-d-Glucogallin in Canarium album L., a Functional Fruit Rich in Hydrolysable Tannins
Source: Molecules. 2021 Jul 31;26(15):4650. doi: 10.3390/molecules26154650 (PMC8347697; doi:10.3390/molecules26154650)
Supplement: Supplementary file 1 [file molecules-26-04650-s001.zip › Supplementary information final 8.2.pdf]

Supplementary information

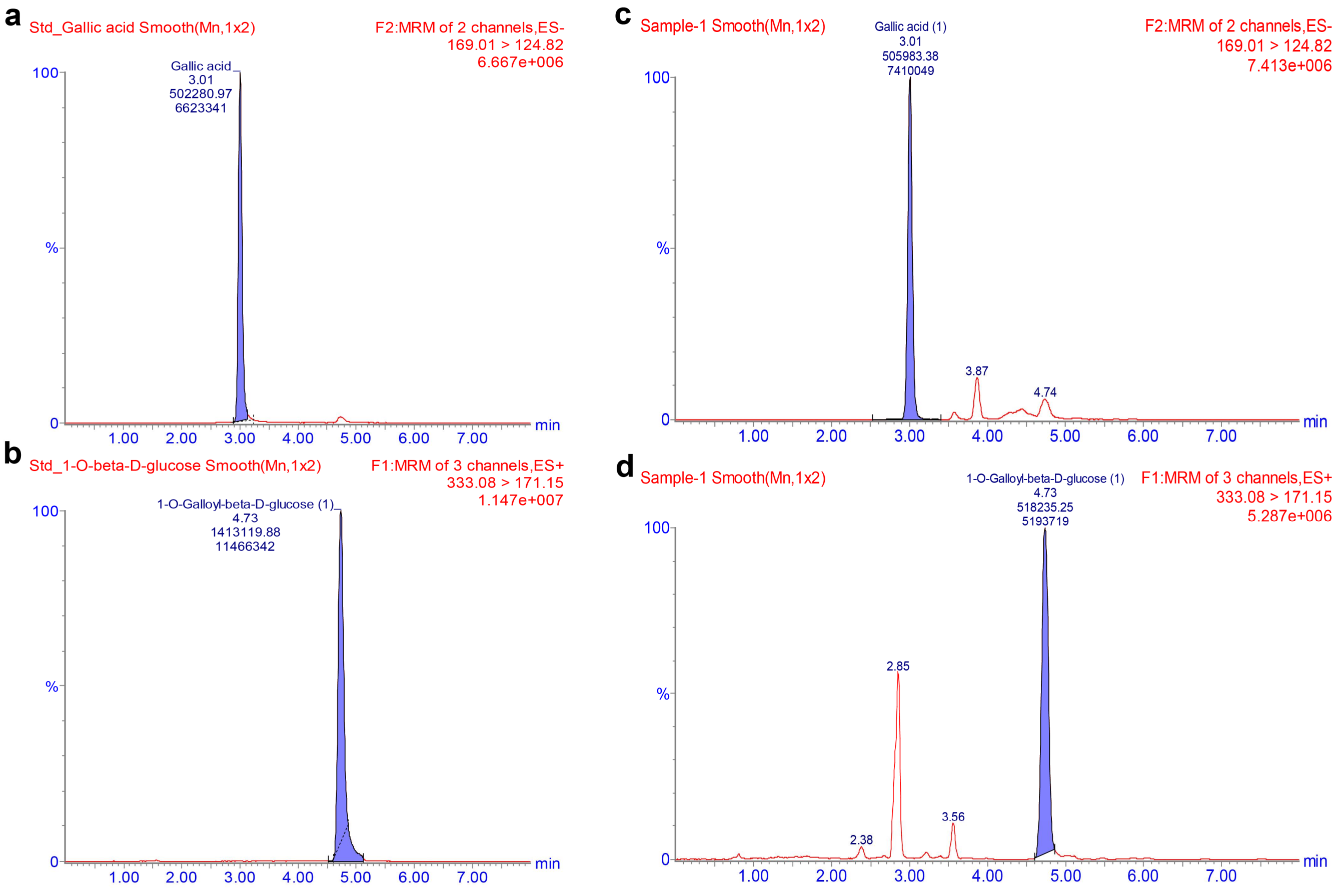

Figure S1. Chromatograms of standards and typical samples. (**a**), (**c**)Chromatograms of GA standard and sample in MRM mode, ESI-, m/z 169.01→124.82, retention time 3.01. (**b**), (**d**)Chromatograms of βG standard and sample in MRM mode, ESI+, m/z 333.08→171.15, retention time 4.73.

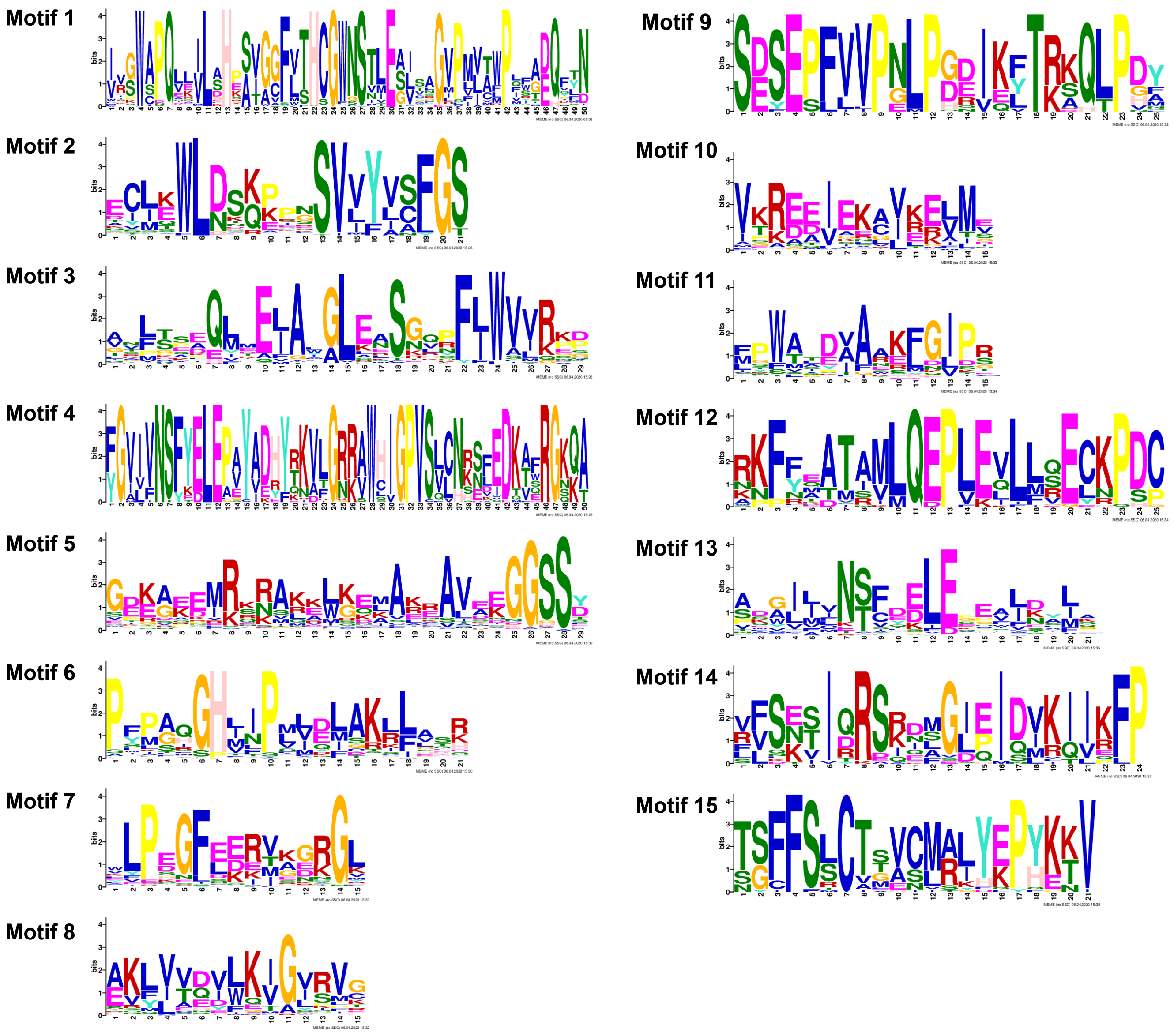

Figure S2 Conserved motifs from 140 CaUGTs. All 15 motifs were identified by MEME online software (http://meme-suite.org/).

```
                10        20        30        40        50        60        70        80        90        100
1      ATGGGCTCTGAGTCTCTTGTCCATGTCTTGCTTGTTTCATTCCCTGGCCAAGGCCACGTGAACCCTCTTCTTAGACTTGGCAAGAAACTAGCTTCAAAGGGTTTG
1       M  G  S  E  S  L  V  H  V  L  L  V  S  F  P  G  Q  G  H  V  N  P  L  L  R  L  G  K  K  L  A  S  K  G  L

               115       125       135       145       155       165       175       185       195       205
106    CTTGTCACCTTCACCACTCCTGAAACCACCGGCAAACAATTGAGGAAAGCTAGCAACGCCTCCGAAGAGCCGACCCCGATTGGAGATGGCTACATTCGGTTCGAA
36      L  V  T  F  T  T  P  E  T  T  G  K  Q  L  R  K  A  S  N  A  S  E  E  P  T  P  I  G  D  G  Y  I  R  F  E

               220       230       240       250       260       270       280       290       300       310
211    TTCTTCGAAGATGGTTGGGATGAGGATGAGCCAAGGCGCCAAGACCTTGACCAATACTTGCCTCAACTAGAGTTGATTGGCAAAGATGTGATTCCACGTATGATC
71      F  F  E  D  G  W  D  E  D  E  P  R  R  Q  D  L  D  Q  Y  L  P  Q  L  E  L  I  G  K  D  V  I  P  R  M  I

               325       335       345       355       365       375       385       395       405       415
316    CAGAGAAACGCAGAACAAAACCGCCCCATTTCTTGCCTAATCAACAATCCTTTCATTCCTTGGGTATCCGACGTCGCTACAAGTCTTGGCCTCCCTTCTGCAATG
106     Q  R  N  A  E  Q  N  R  P  I  S  C  L  I  N  N  P  F  I  P  W  V  S  D  V  A  T  S  L  G  L  P  S  A  M

               430       440       450       460       470       480       490       500       510       520
421    CTTTGGGTCCAATCCTGCGCGTGTTTCTCCTCTTATTATCATTACTATCACGGGTTGGTGCCGTTTCCAAGTGAAGAACACCCTGAAATCGATGTTCAGTTGCCT
141     L  W  V  Q  S  C  A  C  F  S  S  Y  Y  H  Y  Y  H  G  L  V  P  F  P  S  E  E  H  P  E  I  D  V  Q  L  P

               535       545       555       565       575       585       595       605       615       625
526    TGTATGCCCCTCCTGAAGTACGATGAAGTTCCAAGCTTCTTGCATCCGACAACTCCTTACCCTTTCCTGAGGAGAGCTATTCTCGGTCAGTACAGAAACCTTGAC
176     C  M  P  L  L  K  Y  D  E  V  P  S  F  L  H  P  T  T  P  Y  P  F  L  R  R  A  I  L  G  Q  Y  R  N  L  D

               640       650       660       670       680       690       700       710       720       730
631    AAGCCTTTTTGCATACTGATGGAAAGTTTCCAAGAGCTTGAGCCTGAGGTCATCGAATACATGTCAAAGGTTTGTCCAATCAAGACAGTTGGCCCACTATTCAAG
211     K  P  F  C  I  L  M  E  S  F  Q  E  L  E  P  E  V  I  E  Y  M  S  K  V  C  P  I  K  T  V  G  P  L  F  K

               745       755       765       775       785       795       805       815       825       835
736    AACCCTAAAGCGCCAACCTCAACTGTCCGCGGTGACCTCATGCAGGCCGATGATTGCATCGAGTGGCTAGACAGTAAACCTCCTTTCTCCGTCGTTTACATCTCT
246     N  P  K  A  P  T  S  T  V  R  G  D  L  M  Q  A  D  D  C  I  E  W  L  D  S  K  P  P  F  S  V  V  Y  I  S

               850       860       870       880       890       900       910       920       930       940
841    TTCGGCAGCGTCGTTTACCTGAAGCAAGAACAAATAGACGAAATCGCTTATGGCTTACTGAACTCTGGAGTTTCGTTCTTGTGGGTGATGAAGCCGCCGCATAAA
281     F  G  S  V  V  Y  L  K  Q  E  Q  I  D  E  I  A  Y  G  L  L  N  S  G  V  S  F  L  W  V  M  K  P  P  H  K

               955       965       975       985       995      1005      1015      1025      1035      1045
946    GATTCTGGCTACGAACTTAATGTTCTCCCAGATGGGTTCCTGGAGAAAGTTGGAGACAACGGCAAGGTCGTAAAATGGAGTCCACAGGAGAAGGTCTTAGCACAC
316     D  S  G  Y  E  L  N  V  L  P  D  G  F  L  E  K  V  G  D  N  G  K  V  V  K  W  S  P  Q  E  K  V  L  A  H

              1060      1070      1080      1090      1100      1110      1120      1130      1140      1150
1051   CCAAGTGTTGCCTGCTTCGTAACTCACTGCGGATGGAACTCAACGATGGAGTCACTGTCGTCAGGGATGCCGGTTGTGGCCTTCCCGCAATGGGGCGATCAAGTC
351     P  S  V  A  C  F  V  T  H  C  G  W  N  S  T  M  E  S  L  S  S  G  M  P  V  V  A  F  P  Q  W  G  D  Q  V

              1165      1175      1185      1195      1205      1215      1225      1235      1245      1255
1156   ACTGACGCTGTGTACTTGGTGGACGTGTTCAAGACTGGAATAAGAATGTGCCGTGGAGAGGCAGAGAACAGGGTGATTCCCCGTGATGAGATTGAGAAATGCTTG
386     T  D  A  V  Y  L  V  D  V  F  K  T  G  I  R  M  C  R  G  E  A  E  N  R  V  I  P  R  D  E  I  E  K  C  L

              1270      1280      1290      1300      1310      1320      1330      1340      1350      1360
1261   CTTGAGGCCACTTCCGGGCCCAAGGCTGCAGAGATGAAGCAAAATGCATTGAAGTGGAAGAAAGCGGCGGAGGAAGCGGTGGCCGAAGGTGGCTCCTCCGATAGT
421     L  E  A  T  S  G  P  K  A  A  E  M  K  Q  N  A  L  K  W  K  K  A  A  E  E  A  V  A  E  G  G  S  S  D  S

              1375      1385      1395      1405      1415      1425      1435      1445      1455      1465
1366   AACATCCAAGCGTTTGTTGACGAGGTGAGAAGGAGAAGTGTTGAAATTCATTGCAAGTCAAAGGCCATCAAGCCAACAACAGCCAATGGAGTTGCTGATTTGGTC
456     N  I  Q  A  F  V  D  E  V  R  R  R  S  V  E  I  H  C  K  S  K  A  I  K  P  T  T  A  N  G  V  A  D  L  V

              1480      1490      1500      1510
1471   GAGAAGGCAGCCAATGGGAAAGTAGAATTGGTGGAGTCCTGA
491     E  K  A  A  N  G  K  V  E  L  V  E  S  *
```

Figure S3 Coding sequence and encoded amino acid sequence of *UGT84A77*. ATG, start codon; *, stop codon; the underlined part indicates the conserved UDPGT domain; the shaded part indicates the PSPG box; ▲ indicates the UDPG recognition site.

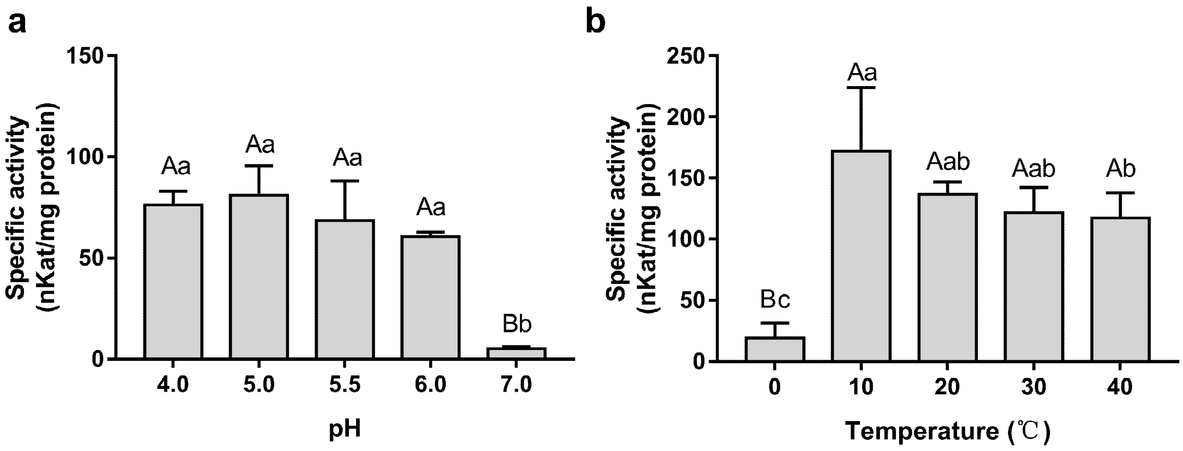

Figure S4 Effects of pH (**a**) and temperature (**b**) on enzyme activity of UGT84A77 recombinant protein.

Table S1 Information of UGTs for phylogenetic analysis

Species	Gene	GenBank accession	Reference
Canarium album L.	140 UGTs		This study
Arabidopsis thaliana	122 UGTs	See reference	http://www.p450.kvl.dk/UGT.shtml
Prunus persica	Prupe.7G055200	ONH95178.1	(Cheng et al. 2019)
	Prupe.6G265900	ONI03570.1	
	Prupe.6G267000	ONI03581.1	
	Prupe.6G266600	ONI03577.1	
Zea mays	GRMZM2G075387	ONM25949.1	
	GRMZM2G168474		
	GRMZM5G834303		
Punica granatum	PgUGT95B2	MH507175.1	
	PgUGT84A23	KT159805.1	(Ono et al. 2016a)
	PgUGT84A24	KT159807.1	
Quercus robur	QrUGT84A13	KF527849.1	(Mittasch et al. 2014)
Fragaria × ananassa	FaGT2	AY663785.1	(Schulenburg et al. 2016)
Vitis vinifera	VvGT1	JN164679.1	(Khater et al. 2012)
	VvGT2	JN164680.1	
	VvGT3	JN164681.1	
Camellia sinensis	CsUDT84A22	ALO19890.1	(Cui et al. 2016b)

Table S2 Primer sequences of the 12 *CaUGTs* for RT-qPCR

Gene ID	Sense primer sequence (5'-3')	Anti-sense primer sequence (5'-3')
Isoform0117852	TTGTCACCTTCACCACTCC	CATCCTCATCCCAACCAT
Isoform0027939	TATGGAGGAGAAAAAAGCG	TGACTATGTATTGATCGTGGAG
Isoform0016456	ATGATGACACGGGCAACTA	TAACCACTCCAGGCAACC
Isoform0026341	GGTAGGATGATAGAGGCTGTG	TCCTCATTTTCTTCCCTTGT
Isoform0033289	GGTAGGATGATAGAGGCTGTG	TCCTCATTTTCTTCCCTTGT
Isoform0025823	GAAGTTGAGGCTGAAAAGAT	TGAAGCAGACCAAAAGGA
Isoform0012535	ACAATCAAAGCCAAAGCAG	TGGAAATACAACTCGGAAAC
Isoform0118359	ACGAAGTTGAGGCTGAAAA	TTGAAGCAGACCAAAAGGA
Isoform0011864	GGCTCGGTGTTATTCGTCT	CTTATCATTTGGGCTTCTCA
Isoform0117204	CAAAAGGCTAAGAAGGAAGA	CACTCACAAACTTGGCACTA
Isoform0029193	CTGCTGCTATGGAGTATGCT	GACCAAGAGACCGTCGTTT
Isoform0121163	CTACCCTTGTTGTTTTCTCCT	CAGTTGTCTTTTGCTATCCG
CaACT7	AACCCCAAGGCTAACAGG	TGGCATACAGAGACAGGACA

Table S3 Comparison of enzyme kinetic parameters of the UGT84A subfamily in different species

Species	Enzyme	Substrate	Km (μM)	Kcat (s^{-1})	Kcat/Km ($s^{-1}\cdot M^{-1}$)	Reference
Canarium album	UGT84A77	GA	108.90 ± 21.06	3.71 ± 0.18	34076.64	This research
		UDPG	193.30 ± 34.33	0.74 ± 0.04	3844.87	
Vitis vinifera	VvgGT1	Gallic acid	510±32	2.16±0.28	4231	Khater et al. 2012
		Caffeic acid	165±67	1.16±0.12	7069	
	VvgGT2	Gallic acid	884±188	2.82±0.24	3194	

		Caffeic acid	105±30	1.48±0.09	14142	
	VvgGT3	Gallic acid	566±190	2.19±0.09	3873	
		Caffeic acid	142±57	1.07±0.10	7567	
Eutrema japonicum	UGT84A57 (WjGT1)	Apigenin	1.28 ± 0.41	0.55 ± 0.09	4.4×10^5	(Mashima et al. 2019)
		UDP-glucose	168 ± 38	0.15 ± 0.02	8.9×10^2	
Eucalyptus camaldulensis	UGT84A25a	Gallic acid	168 ± 14	13.5 ± 0.3		(Tahara et al. 2018a)
		Protocatechuic acid	170 ± 9	12.7 ± 0.2		
		Vanillic acid	103 ± 8	12.6 ± 0.2		
	UGT84A26a	Gallic acid	190 ± 16	10.7 ± 0.2		
		Protocatechuic acid	149 ± 7	10.1 ± 0.1		
		Vanillic acid	111 ± 9	10.4 ± 0.2		
Quercus robur	UGT84A13	Gallic acid	420			(Mittasch et al. 2014)
		Protocatechuic acid	290			
		Vanillic acid	230			
Camellia sinensis	CsUGT84A22	Gallic acid	758.4			(Cui et al. 2016a)
		p-Coumaric acid	531.4			
Punica granatum	UGT84A23	Gallic acid	890±70	0.52±0.03	580	(Ono et al. 2016b)
		4-hydroxy benzoic acid	1190±30	0.61±0.03	510	
		3,4-dihydroxy benzoic acid	2460±260	0.76±0.06	310	
		Catfeic acid	1060+110	0.64±0.05	600	
		Cinnamic acid	1120±80	0.56±0.02	500	
		Coumanic acid	940±130	0.52±0.07	550	
		Ferulic acid	1580±10	0.72±0.01	460	
		Sinapic acid	860±50	0.5±0.02	580	
	UGT84A24	Gallic acid	980±10	0.55±0.01	560	
		4-hydroxy benzoic acid	1170±30	0.6±0.01	510	
		3,4-dihydroxy benzoic acid	4440±730	1.35±0.19	300	
		Catfeic acid	1770+170	0.89±0.08	500	
		Cinnamic acid	860±90	0.45±0.04	520	
		Coumanic acid	1060+150	0.44±0.08	420	
		Ferulic acid	1320±20	0.66±0.01	500	
		Sinapic acid	780±20	0.44±0.01	560	

Table S4 PCR primer sequences for *UGT84A77*

Primers	Primer sequence (5'-3')
UGT84A77-F	caccATGGGCTCTGAGTCTCTTGTC
UGT84A77-R	GGACTCCACCAATTCTACTTTCC
UGT84A77-F-BamH I	gagagaGGATCCATGGGCTCTGAGTCTCTTGTC
UGT84A77-R-Sal I	gagagaGTCGACGGACTCCACCAATTCTACTTTCC

Note: Lowercase letters denote protective bases, and underlining denotes digestion sites.
